# Supplementary figures and images for: Inhibition of Ribosome Assembly and Ribosome Translation Has Distinctly Different Effects on Abundance and Paralogue Composition of Ribosomal Protein mRNAs in Saccharomyces cerevisiae
Source: mSystems. 2023 Jan 18;8(1):e01098-22. doi: 10.1128/msystems.01098-22 (PMC9948716; doi:10.1128/msystems.01098-22)

### Sucrose gradients

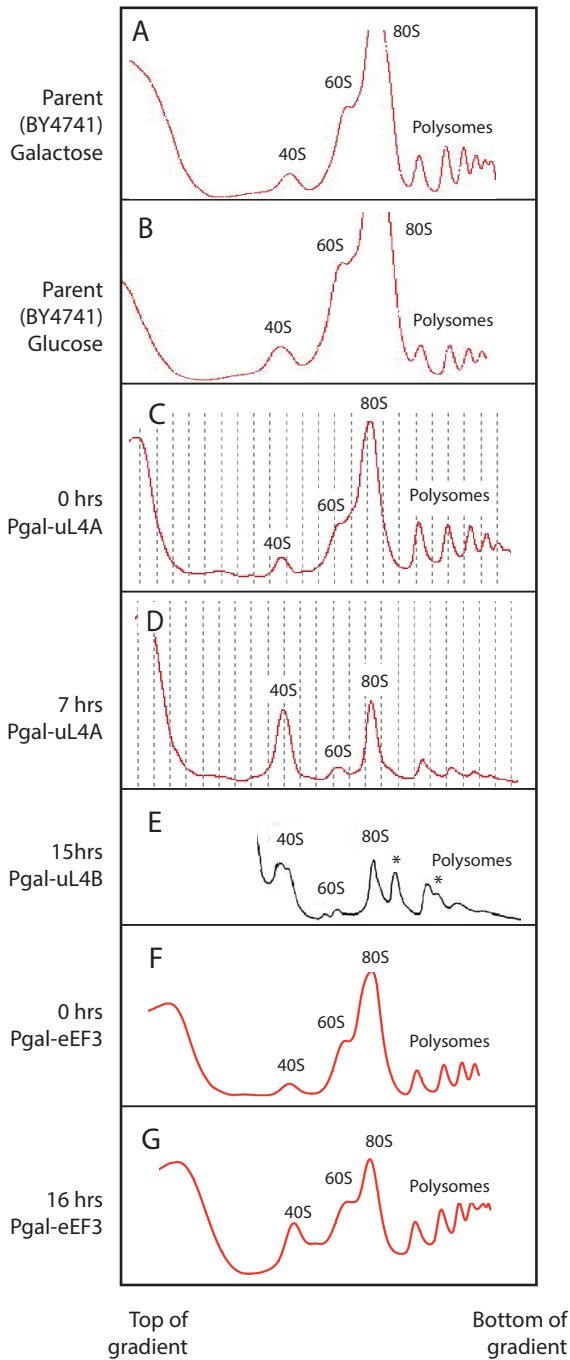

### Northern uL4 mRNA

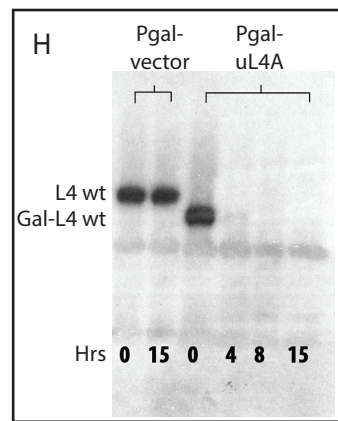

### Western analysis of eEF3

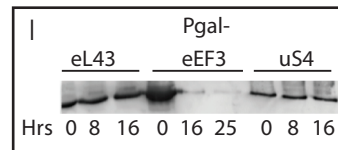

### Growth curve for Pgal-uL4B

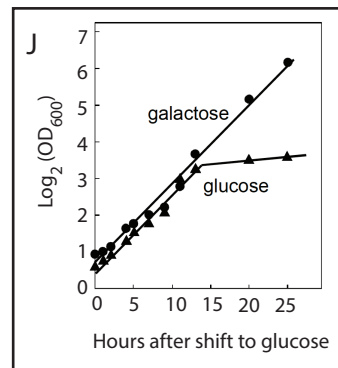

Supplement: FIG S1 [file msystems.01098-22-s0001.pdf]

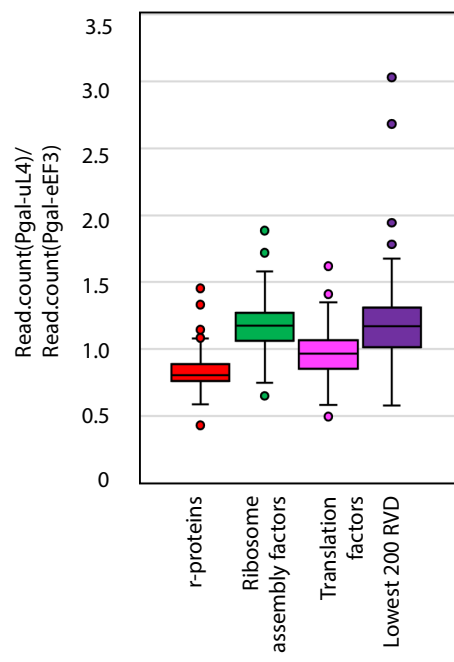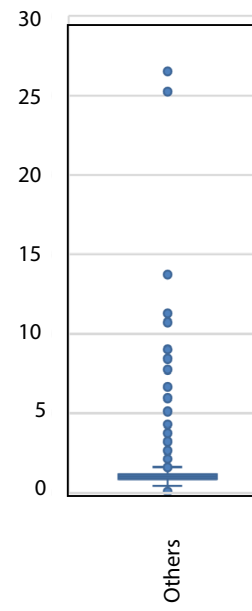

Supplement: FIG S3 [file msystems.01098-22-s0003.pdf]

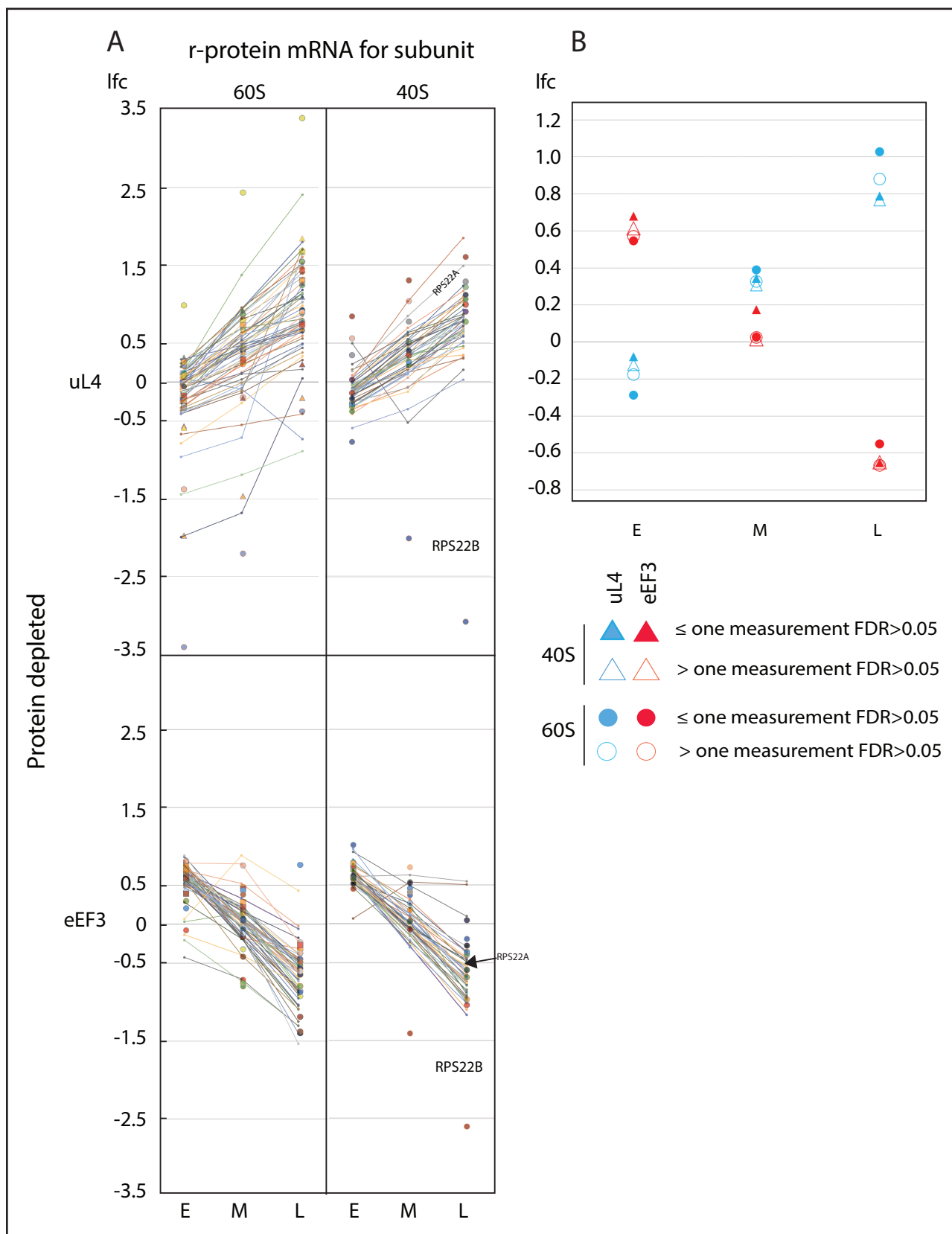

Supplement: FIG S2 [file msystems.01098-22-s0002.pdf]
