# Supplementary material for: Inhibition of Ribosome Assembly and Ribosome Translation Has Distinctly Different Effects on Abundance and Paralogue Composition of Ribosomal Protein mRNAs in Saccharomyces cerevisiae
Source: mSystems. 2023 Jan 18;8(1):e01098-22. doi: 10.1128/msystems.01098-22 (PMC9948716; doi:10.1128/msystems.01098-22)
Supplement: TABLE S1 [file msystems.01098-22-s0004.pdf]

Table S1. Strains used

Strain name	Gene expressed from the *GAL1/10* promoter	Protein expressed from *GAL1/10* promoter	Genotype	Reference
BY4741	None	None	Matα; his3Δ1; leu2Δ0; lys2Δ0; ura3Δ0	(56)
Pgal-uL4A (JWY8402)	RPL4A	uL4A	ura3-52; trp1-101; lys2-801; his3-d200/leu2- d1; rpl4b::KANMX6; GAL-3HA-RPL4A (TRP1)	(57)
Pgal-eEF3 (YLL1131)	*TEF3* alias *YEF3*	eEF3	his3Δ1; leu2Δ0; lys2Δ0; ura3Δ0; GAL1/10-TEF3/YEF3 (KanMX)	(26)
Pgal-uL4B (YLL2083)	*RPL4B*	uL4B	ade2-101; his3A200; ura3-52; lys2; rpl4AΔ::KAN rpl4BΔ::lox / pGAL-RPL4; HIS3	This paper
